# Adapted CBT to Stabilize Sleep on Psychiatric Wards: a Transdiagnostic Treatment Approach

**DOI:** 10.1017/S1352465817000789

**Published:** 2018-04-04

**Authors:** Bryony Sheaves, Louise Isham, Jonathan Bradley, Colin Espie, Alvaro Barrera, Felicity Waite, Allison G. Harvey, Caroline Attard, Daniel Freeman

**Affiliations:** 1 Sleep and Circadian Neuroscience Institute, Department of Psychiatry, University of Oxford, Oxford, UK; 1a Oxford Health NHS Foundation Trust, Oxford, UK; 2 Oxford Health NHS Foundation Trust, Oxford, UK; 2a Sleep and Circadian Neuroscience Institute, Department of Psychiatry, University of Oxford, Oxford, UK; 3 Sleep and Circadian Neuroscience Institute, Department of Psychiatry, University of Oxford, Oxford, UK; 3a Oxford Health NHS Foundation Trust, Oxford, UK; 4 Sleep and Circadian Neuroscience, Nuffield Department of Clinical Neurosciences, University of Oxford, Oxford, UK; 5 Oxford Health NHS Foundation Trust, Oxford, UK; 5a Department of Psychiatry, University of Oxford, Oxford, UK; 6 Sleep and Circadian Neuroscience Institute, Department of Psychiatry, University of Oxford, Oxford, UK; 6a Oxford Health NHS Foundation Trust, Oxford, UK; 7 University of California, Berkeley, USA; 8 Berkshire Health NHS Foundation Trust, Berkshire, UK; 9 Sleep and Circadian Neuroscience Institute, Department of Psychiatry, University of Oxford, Oxford, UK; 9a Oxford Health NHS Foundation Trust, Oxford, UK

**Keywords:** sleep, insomnia, inpatient, ward, psychosis, bipolar disorder

## Abstract

**Background:** Almost all patients admitted at acute crisis to a psychiatric ward experience clinically significant symptoms of insomnia. Ward environments pose challenges to both sleep and the delivery of therapy. Despite this, there is no description of how to adapt cognitive behavioural therapy (CBT) for insomnia to overcome these challenges. **Aims:** (i) To describe the key insomnia presentations observed in the Oxford Ward Sleep Solution (OWLS) trial and (ii) outline key adaptations aimed to increase accessibility and hence effectiveness of CBT for insomnia for a ward setting. **Methods:** Trial therapists collaboratively agreed the key insomnia presentations and therapy adaptations based on their individual reflective logs used during the trial. **Results:** Three key insomnia presentations are outlined. These are used to illustrate the application of 10 CBT for insomnia therapy adaptations. These include use of sleep monitoring watches to engage patients in treatment, stabilizing circadian rhythms, reducing the impact of night-time observations and managing discharge as a sleep challenge. **Conclusions**: Whilst inpatient wards bring challenges for sleep and therapy delivery, creative adaptations can increase the accessibility of evidence based CBT for insomnia techniques. This therapy has proven popular with patients.

## Introduction

Difficulties getting to or staying asleep (symptoms of insomnia) are almost ubiquitous on psychiatric inpatient wards. Despite this there has been no description of evidence-based psychological approaches to treat insomnia for acutely unwell hospitalized patients. Medication has been the traditional treatment approach. However, even with their sedative effects, sleep disturbance remains pervasive (Waters et al., 2012). Evidence from community settings indicates that adapted cognitive behavioural therapy (CBT) treatment protocols (Kaplan and Harvey, 2013; Waite et al., 2015) may be highly effective for treating insomnia for patients with psychosis (Freeman et al., 2015b) and bipolar disorder (Harvey et al., 2015).

The Oxford Ward Sleep Solution (OWLS) pilot randomized controlled trial recruited forty patients admitted to a psychiatric inpatient ward. Each was experiencing symptoms of insomnia and was randomly allocated to receive adapted CBT to stabilize sleep, in addition to standard care, or standard care alone. The psychological intervention for sleep, tailored to an acute care setting, led to quicker and potentially fuller recovery of insomnia than standard care alone (Sheaves et al., 2017). The treatment effect on insomnia, as assessed by the Insomnia Severity Index (Bastien et al., 2001) was large (*d* = 0.9) with promising results for psychological wellbeing. Those in the sleep treatment group were discharged on average 8.5 days earlier than those receiving standard care alone. Whilst this is a pilot, and further investigation is required, there may be economic as well as clinical benefits to this approach.

The current paper outlines key insomnia presentations and the therapeutic approach. Following this, ten treatment adaptations for delivering CBT for insomnia with an inpatient population will be summarized using illustrative case examples (Table 1). The therapist's rationale for each technique is outlined, just as it was shared with patients.

**Table 1.** Three key insomnia presentations in the OWLS (inpatient ward) trial

**Anxiety-related hyperarousal at night**	
Billy* was admitted during a first episode of psychosis. He spent much time in his hospital bedroom worrying about his paranoid belief that others were out to attack him. He was concerned that staff and patients were part of the conspiracy. He also worried about the impact of hospitalization on his college course and spent time in his bedroom trying to work (particularly at night when the ward was quieter). Both worry and working left him feeling more alert at night, and he reported that it could take him up to 4 hours to get to sleep. Being unable to sleep at night afforded him more time to worry about others which exacerbated his delusional belief. Understandably he also had difficulties getting up in the morning and felt fatigued throughout the day. This left him with fewer resources to cope with the stressors of being in hospital which in turn exacerbated his anxiety.	
This group is characterized by symptoms of insomnia, driven by worry and resultant anxiety. This causes pre-sleep hyperarousal which disrupts sleep. However, in contrast to the patient with insomnia without co-morbidities, the content of worry is rarely about sleep but, rather, related to illness, concerns about being detained in hospital, and the consequences of this for life at home (e.g. relationships, accommodation and family life). Consequently, patients often have a low sleep efficiency (i.e. a low proportion of the time in bed is spent asleep).	
**Mania-related hyperarousal at night**
Sandeep* was admitted to the ward in the midst of an acute manic episode. On average he was achieving 2‒3 hours of fragmented sleep per night, but some nights no sleep at all. He reported feeling energized at night and wanted to use this energy to work on multiple projects. During the night he stayed up working or spent time in the communal areas of the ward initiating animated discussions with staff and other patients. Sandeep found staff suggestions to return to his room very frustrating due to his energy levels and this could result in disagreements. During the day Sandeep was active but by late afternoon or early evening sometimes ‘crashed’ – napping for an hour or so. This made it even harder for him to wind down at night, and fed into a vicious cycle of sleep disruption.	
This group is characterized by a perceived reduced need for sleep occurring within the context of mania. At the start of the usual sleep window, patients are alert, active and energized (and hence not sleepy). As with the insomnia group, this is driven by hyperarousal; however, in this group, the driving force is typically positive affect or irritability, rather than worry. The patient response is to take advantage of this additional energy by doing stimulating activities (e.g. work, exercise and interacting energetically with others), rather than winding down for sleep. This activates them further and means that individuals often have only a few hours, or sometimes no sleep at night. Naps in the day are common, which reduce the likelihood of sleep later that night. For some, sleep deprivation eventually triggers a period of vast oversleeping (e.g. 12 hours), which subsequently reduces the likelihood of night-time sleep the following night.	
**Circadian rhythm (body clock) disruption**	
Zak* was admitted to hospital during a severe depressive episode, which had worsened in the context of family difficulties and homelessness. In addition to very low mood, he reported experiencing slowed thinking, extreme fatigue and spent most of his time in his bedroom sleeping intermittently. As a result he did not attend ward rounds or occupational therapy groups. He reported that he liked sleep because it was an escape from his low mood. At night he struggled to initiate and maintain sleep, despite hypnotic medication. He was typically in bed for 20 hours per day and estimated sleeping for around 14 of these. He used coffee and energy drinks to try to combat daytime fatigue.	
This group is characterized by an ill-defined sleep‒wake cycle. This includes sleeping in the daytime (and being awake at night), going to bed early and waking early (phase advance), sleeping late at night and into the morning (phase delay) or having no regular pattern to sleep and wakefulness (free running). This presentation is accompanied by an inability to initiate or maintain sleep at the desired sleep time or non-restorative sleep. However, when assessing total sleep time, often patients are sleeping for a long time (hypersomnia). The consequences of circadian rhythm disruption included missing out on meal times and therapeutic opportunities and having reduced sleep quality due to daylight and noise.	

*Names given are pseudonyms. Some details are altered to retain confidentiality.

### Description of the sample and key insomnia presentations

Patients were recruited from one male acute psychiatric inpatient ward in Oxford Health NHS Foundation Trust (UK). Each had self-reported symptoms of insomnia, and psychiatric symptoms. The most common diagnoses were psychoses, bipolar affective disorder and depression. The impact of individual psychiatric symptoms on the sleep and circadian system, and implications for treatment delivery are summarized in Table 2. The therapists described the insomnia of each participant based on the maintenance formulation. Similarities were observed across participants which clustered into three key presentations, illustrated using case examples (Table 1). Whilst distinct, these presentations were not mutually exclusive.

**Table 2.** The impact of psychiatric symptoms on the sleep and circadian system, and implications for treatment

Psychiatric symptom	Impact of psychiatric symptom on sleep and circadian system	Treatment adaptation
Mania	Hypersensitivity to light disrupting circadian rhythm	Emphasis on very low level light during wind-down routine
	Excess energy across the day	Graded wind-down routine
Heightened activity in pre-sleep period fuelling wakefulness	Use of fitness tracker to agree upper limit on step count, particularly in the evening
Depression	Increase in perceived need for sleep due to fatigue	Psychoeducation about link between hypersomnia and fatigue
	Oversleeping/napping reducing night time sleep pressure	Energy experiments to discover that activity rather than sleep generates more energy (if oversleeping)
	Poverty of daytime activity increasing opportunity to nap	Light exposure to boost daytime arousal
Increased use of caffeine/energy drinks across the day	Use of caffeine limited to the morning
Anxiety	Heightened worry fuelling pre-sleep hyperarousal	Greater emphasis on wind-down, relaxation and worry management strategies
Auditory hallucinations	Voices delaying sleep onset	Wind-down and relaxation to target voices triggered by heightened affect
	Stimulus control to break the association between bed and hearing voices
Persecutory delusions	Heightened worry increasing sense of threat which fuels pre-sleep hyperarousal	Strategies to increase sense of safety, e.g. coping cards, safe place imagery, worry management strategies
Disrupted circadian rhythm	Light and activity scheduling to regulate circadian rhythm

### Overview of therapy: a targeted high-intensity approach

The goal of therapy was to stabilize sleep to promote recovery. The sleep treatment at acute crisis consisted of three key factors: (i) sleep and activity monitoring devices for assessment and to engage patients in their treatment, (ii) CBT for insomnia, and (iii) enhanced light exposure. The mean number of sessions received was nine, delivered over a 14-day window. This is brief, focused, and highly intensive therapy. The mean duration of sessions was 44.8 minutes (standard deviation 15.6). Key targets included: (i) optimizing homeostatic sleep pressure, (ii) stabilizing the circadian rhythm, and (iii) reducing pre-sleep hyperarousal.

A 2-week therapy window is a departure from the typical pacing of psychological therapy but allowed all but one patient to receive a full course of therapy prior to discharge. Meeting patients four to five times per week allowed the sleep plan to be optimized in light of rapid changes in presentation. It also allowed between-session tasks to be supported by swift problem solving prior to the following night of sleep. Clinical supervision was broken into shorter (15 minute) sessions every other day, rather than longer weekly sessions.

The highly collaborative approach, a central hallmark of CBT, was particularly important. Many inpatients receive treatment involuntarily, under a section of the Mental Health Act (MHA) 1983. Hence regular observations form a standard part of care. These necessary processes create a power imbalance, and challenge a collaborative approach. However, the therapy brought opportunities to instil collaboration. The patient's experience of sleep was of central focus (and given greater emphasis than staff perceptions of patients’ sleep) and behavioural experiments within sessions allowed the patient and therapist to discover successful strategies together. The trial therapists were struck by the extent to which patients appreciated and responded to this collaborative approach, and felt that this significantly enhanced engagement and motivation.

## 1. Engagement and assessment

‘*When people sleep better, they tend to feel better*’

This rationale for the intervention was intuitively engaging to patients who viewed sleep as important in its own right and/or as a means to recover from mental health issues. Zak (experiencing depression) wanted to sleep through the night and combat fatigue. In contrast, Sandeep (experiencing mania) was less concerned about sleep loss *per se* but wanted to use sleep to boost daytime performance (e.g. work projects) and recognized that under-sleeping may prolong his admission.

Sleep was a normalizing and inclusive topic which felt safe to discuss. This was important at a time when some patients felt reluctant to talk about other mental health symptoms due to fear of it impacting on their length of admission, exacerbating distress or stigma. For example, Billy was reluctant to talk about his paranoid beliefs for fear that he would be kept in hospital under section longer. However, his difficulty getting to sleep was frustrating and distressing and his goal to improve sleep was shared between him and the staff.

Assessment was focused on maintenance factors for insomnia. The aim was to build a sleep-focused formulation to inform quick use of CBT for insomnia techniques, rather than aiming for a complex formulation of multiple symptoms. However, co-morbid symptoms did feature in the formulation if they were maintaining insomnia (e.g. if depression caused difficulties getting up in the morning). Although the formulation evolved over time (as in all CBT), typically it was advanced enough by the end of the first session to enable intervention strategies to be implemented immediately. A tick list of key factors that commonly maintain insomnia facilitated this (see Table 3).

**Table 3.** Tick-list used with patients to assess common maintenance factors for insomnia on an acute inpatient ward (delivered after sleep psycho-education)

	*Tick if this is relevant to you*
I nap in the day, which decreases my ‘sleep pressure’ at night	□
I go to bed early which decreases my ‘sleep pressure’	□
I lie in in the mornings and can't sleep the following night	□
I don't have a regular bed time and wake-up time	□
My room is too light	□
I don't feel relaxed when I go to bed	□
I feel worried about sleeping	□
I hear voices that stop me sleeping	□
I'm woken up by ward lights or noises	□
I don't do much in the day, so am not tired enough to sleep well	□
I do things in the evening which make me feel more alert rather than sleepy (e.g. watch a scary film, doing exercise)	□
I don't have a regular pre-bed routine that helps me unwind	□
I drink caffeine in the afternoon/evening	□
I'm too hot/cold at night	□
I smoke before going to bed	□
My bed is uncomfortable	□
I take medication or substances which may affect my sleep	□

## 2. Wearable devices for assessment and engaging patients in their treatment

‘*The watch can help us learn about your sleep. We can look at the data together and make sure that the strategies we come up with are most likely to work for you.*’

Patients were offered use of a sleep and activity monitoring watch during therapy. This device combines heart rate and movement data to infer sleep periods. It was introduced with a clear rationale for use, a demonstration of its functions, and patients could choose whether or not to use it. This transparency allowed all patients, including those with severe paranoia, to see a purpose to the watch. Consequently, all patients took the opportunity to use one.

At the start of therapy, watches were used to assess sleep timings, the consistency of sleep and rise times across nights, daytime naps and circadian rhythms. This was an effortless way of gaining rich assessment information, gave valuable clues about which sleep goal may be realistic (e.g. a realistic sleep window to aim for) and which strategies were likely to be effective. At the end of therapy discussing improvements in sleep (evidenced by watch data), and linking these back to the techniques used enabled patients to recognize effective strategies. Watch data were shared in graphs with patients to celebrate sleep improvement and these were integrated into end-of-therapy letters.

For Sandeep, the activity data highlighted a circadian phase delay whereby heightened activity in the pre-sleep period shifted the sleep window later. This informed the timing of activity scheduling (boosting activity in the morning and decreasing it in the evening) to increase the chances of sleep at the goal times. He also watched the instant heart rate feedback during relaxation which helped to anchor his attention to avoid distraction. Seeing his heart rate drop as a result of relaxation reinforced his efforts.

For Zak, the watch revealed that he lacked a regular sleep‒wake rhythm and spent long periods in bed with long but fragmented sleep periods. The very low step count in the day highlighted the lack of differentiation between day and night. Key strategies were implemented on the basis of this assessment including setting clear wind-down and rise times, reducing naps in the day and boosting daytime activity levels. Achievable daily step count goals were set collaboratively (gradually increasing from 3000 steps per day up to 10,000 by the end of the therapy window). The visible step count offered instant feedback on progress throughout the day, which reinforced his efforts outside of therapy sessions.

Whilst the watches were very informative, it was acknowledged that they were not infallible. The patient perspective on their sleep was prioritized. Where there was a discrepancy between watch data and patient report the possible reasons for this were discussed. For example, therapist: ‘*why do you think the watch could have got it wrong?*’, patient: ‘*I was lying in bed quite still, trying to sleep*’. These discrepancies provided new learning (for example, the amount of time spent in bed awake) and therefore informed treatment (such as use of stimulus control).

## 3. Setting a consistent sleep window

‘*How much sleep do you need each night? Let's match this sleep window to your natural body clock*’

Interventions that increase sleep pressure at night (e.g. sleep restriction, SR) are widely regarded as one of the most powerful ingredients of CBT for insomnia (Miller et al., 2014). Strict SR involves reducing the patient's time in bed to match the current average total sleep duration (e.g. 5 hours). Once a consistent pattern of initiating and maintaining sleep has been established, the sleep window is gradually increased. Whilst effective as a stand-alone treatment strategy (Miller et al., 2014), it is associated with short-term sleep deprivation, increased fatigue and impaired vigilance (Kyle et al., 2014). Within the inpatient population we anticipated that strict SR would be less tolerable, could result in disengagement from therapy and possibly exacerbate other symptoms. We therefore followed the principles (by increasing sleep pressure), but adapted the approach to mitigate these concerns.

We collaboratively set a consistent sleep window, without restricting sleep below the target duration (frequently 7‒8 hours). We optimized the chances of sleep by aligning the window to the circadian rhythm. We asked the patient: ‘*How much sleep do you need when you are at your best/things are going well?*’ and ‘*Are you a morning person, evening person, or neither?*’. The aim was to establish a consistent sleep window that was realistic, and achievable. For Zak (sleeping 14 hours per night and in bed for 20) his goal was to sleep for 9 hours between 12 and 9 a.m. This involved reducing the sleep window and emphasizing the importance of a consistent routine. This was achieved by planning engaging activities allowing him to discover that it was manageable to be awake for longer. Sandeep explained that when at home he usually slept for 7 hours. However, at the start of therapy he was sleeping 2‒3 hours total per night in the context of mania. His goal remained 7 hours, which was gradually achieved by increasing the chances of sleep each night using (i) a sleep window matched to his circadian preference (evening type, hence 2 a.m. start), (ii) graded wind-down, and (iii) dark evening environment.

Daytime napping was common, owing to fatigue (from symptoms or medication) and boredom inherent in any hospital admission. Patients were encouraged to save sleep for their planned sleep window and thereby encourage the build-up of sleep pressure throughout the day, which increases the chance of good quality sleep at night.

Embedded within this work was psychoeducation about the association between both long and short sleep duration and fatigue (‘*finding the right amount of sleep for you*’). The timing of medication was discussed for its sedative effects. Liaison with ward staff ensured that medication was timed to promote the desired sleep onset time, whilst ensuring that the sedative effects did not prohibit waking the following morning. Where there was significant morning sedation, a longer sleep window was considered to take this into account. An evening wind-down and morning rise plan was critical to achieving the planned sleep window.

The sleep window was tested by the patient. Where they continued to experience significant symptoms of insomnia (increased sleep onset latency or wake after sleep onset) either a shorter sleep window, or moving the sleep window (earlier or later) was considered.

## 4. Stimulus control

‘*When we have difficulty sleeping, bed can become associated with wakefulness, anxiety, paranoia or a place where we hear voices. To get sleep back on track, we need to boost the connection between bed and sleep*’

In insomnia, bed can quickly become associated with lying awake for long periods (either when trying to sleep or after waking in the night). For example, Billy described bed being associated with being awake and worrying about others intending to harm him.

The aim of stimulus control (SC) is to break this association by building an alternative association between bed and sleep. This is achieved through three steps: (1) doing activities other than sleep (e.g. reading) outside of the bedroom, (2) only going to bed when feeling sleepy-tired (e.g. yawning, eyes itching), and (3) once in bed, if unable to sleep for more than 15 minutes, getting out of bed and winding down elsewhere until sleepy-tired. SC is associated with a rapid and significant reduction in sleep onset latency (Espie et al., 1989) and is a core component of CBT for insomnia protocols (Espie, 2010).

SC is, however, more challenging in a ward environment. The only private space is the bedroom and patients may not want to spend time in communal areas. For example, due to Billy's paranoia about others, spending time outside of his bedroom was distressing. The communal environment is arousing (including at night); there is opportunity to interact with others, bright lighting, and the patient does not have access to their usual home comforts that are conducive to winding down. This was particularly relevant for patients experiencing mania, who are easily activated with minimal stimuli. In relation to step 2, patients may struggle to only go to bed when sleepy-tired, due to feelings of boredom or distress. This was the case for Zak who, in the context of depression, used bed as an escape.

SC was facilitated by introducing a beanbag to participants’ rooms. This allowed patients to remain in their room, but gave them an alternative place to sit other than the bed, when doing activities or implementing the 15-minute rule. Beanbags were comfortable (and hence popular with patients), soft and wipeable (and therefore low risk of infection and harm to self or others).

Although the beanbag was the preferred method, an alternative approach to implementing the 15-minute rule was to sit up in bed (adopting a different position) when unable to sleep. A previous study has shown that this approach, which also follows the principles of conditioning, is associated with a reduction in insomnia symptoms (Malaffo, 2006). It was therefore used to increase accessibility of SC to those who struggled to leave the bed completely (e.g. due to very low mood).

## 5. Targeting hyperarousal with a graded wind-down

‘*We need to slow down our mind and body in preparation for sleep*’

CBT for insomnia protocols recommend a wind-down period of 60‒90 minutes prior to the sleep window (Espie, 2010). The aim is to foster feelings of relaxation and to develop a routine which becomes conditioned to cue sleep. Together these increase the chance of sleep each night. This is important in a hospital environment where hyperarousal may be particularly pronounced due to the experience of being detained under the MHA, distressing symptoms and the changeable milieu of the ward environment.

The importance of winding down was described to patients using an analogy of a light switch: ‘*whilst we might ideally like to switch between wake and sleep instantly, instead we work more like a dimmer switch where we have to take time to gradually transition between wake and sleep*’. A detailed wind-down routine was developed with patients. This drew on activities that patients already found relaxing (e.g. reading), putting thought and effort into how these could be made accessible on the ward (e.g. asking a family member to bring items to the ward), and also trying new relaxing activities where appropriate.

Risk assessments permitting, battery powered radios (negating the need for power leads which pose a ligature risk) were offered to deliver relaxation audios. Therapists recorded relaxation exercises and downloaded relaxing music to a USB stick for use during the wind-down.

A graded and longer wind-down was helpful for patients experiencing mania because starting with relaxation was so incongruous with manic hyperarousal that it caused irritation, and hence counterproductively elevated arousal. Alternatively, patients simply chose not to engage in the wind-down if it was perceived as boring. For Sandeep, for example, a graded wind-down period started with talking to others, watching TV, playing board games and then gradually worked towards spending time doing a relaxation exercise or listening to soft music in his room with dim lighting.

In rare cases where a patient was particularly elevated and distractible, the therapist visited the ward and prompted the use of the wind-down plan. This additionally gave the therapist important insights into factors that disrupted the wind-down. For example, leaving a dimly lit bedroom part way through the routine to collect medication involved waiting in a brightly lit corridor, which was re-activating. This issue was easily solved by staff delivering medication to the patient's bedroom.

## 6. Syncing circadian rhythms: enhanced light exposure

‘*Sunlight is the best way to help us feel awake, energized and tells our body that it's daytime (wake time!)*’

Exposure to light (and dark) at the right time provides important cues for sleep and wakefulness. Light is detected in the eye, and projected to the master clock in the brain (the suprachiasmatic nuclei) which syncs the body's 24 hour rhythms. When in hospital patients often spend long periods of time inside. Artificial light is less intense (100‒300 lux) than natural daylight (around 10,000 lux) and hence is less effective at syncing the circadian rhythm. Enhanced light exposure was therefore an important addition to the protocol.

Natural daylight or light therapy boxes were used to enhance light exposure. Natural daylight was preferred because it typically incorporated activity (another circadian zeitgeber). It was also readily available to patients after discharge and hence could establish a long-term routine to promote better sleep. Light boxes, however, were useful for individuals who had difficulty leaving the ward, for example due to severe depression, paranoia or being involuntary patients and not having leave agreed under the MHA. In these cases, the light box was placed 35 cm from the individual and administered 10,000 lux of light for 30 minutes.

Manuals highlight the importance of delivering light exposure at the same time each morning, in order to best sync the circadian rhythm (Wirz-Justice et al., 2013). Delivering light in the morning helped to target difficulties waking up, particularly for those with circadian phase delay (an ‘evening person’), hypersomnia, or morning sedation due to medication. Behavioural experiments were utilized to enable patients to discover that light can improve levels of wakefulness and energy. For Zak (who was depressed and oversleeping), therapy sessions (and therefore light exposure) were scheduled at his desired wake time (9 a.m.). Light exposure was graded, starting with opening the curtains, followed by use of the light box. This progressed to using the ward garden and local park.

Using natural light to increase wakefulness was also a fruitful approach for targeting problematic afternoon napping. A walk or time in the ward garden was planned for the time when naps were most likely.

A dark evening environment was used to cue for night time (and therefore sleep). This included using dimmer switches in bedrooms during the wind-down routine. Eye masks and blackout blinds during sleep aimed to decrease the time to fall asleep and the likelihood of early morning waking. This was important given that observations to manage risk at night often required staff to turn the light on periodically. Promoting a dark evening environment was an accessible technique for patients experiencing a manic episode, and was integrated following promising results from a previous pilot of dark therapy (Barbini et al., 2005).

## 7. Syncing circadian rhythms: activity scheduling

‘*To keep the body clock in sync, we need as many clues about the difference between day and night as possible. Being active is a powerful cue for daytime (wake time). It also tires us out, increasing the chances of sleep later that night.*’

Whilst light is commonly regarded as the most important input into the circadian system, physical activity is also associated with circadian alignment (Joo et al., 2016) and reduces the likelihood of falling asleep outside of the planned sleep window (Bonnet and Arand, 2000). Symptoms of psychosis and bipolar disorder are associated with disrupted circadian rhythms (Krane-Gartiser et al., 2016; Wulff et al., 2012) and on an inpatient ward, restricted leave can further disrupt the opportunity to engage in patients’ usual activities, exacerbating circadian dysregulation.

Our approach was to increase activity at particular times of the day, to regulate the circadian rhythm. Physical activity was planned in the daytime, when arousal levels were low. For ‘evening types’ this tended to be in the morning, whilst for ‘morning types’, this tended to be in the afternoon and early evening. The activity output from the monitoring watches facilitated assessment of circadian rhythms (see supplementary material, Figure 1), where darker colours on the step count output tended to correlate with increased arousal. Once the discrepancy between patient's arousal and goal arousal was clear, activities were planned to reduce the discrepancy. ‘Energy giving’ activities (e.g. exercise) were planned for soon after the desired wake time and ‘wind-down’ activities before the start of the sleep window. Support from the occupational therapy team was crucial in this and provided opportunities for structured activity. Sleep hygiene strategies (e.g. reducing caffeine intake) were also planned within the framework of the circadian rhythm, for example timing a coffee for the morning and avoiding this in the afternoon and evening.

Timing activities was particularly effective for patients with mood disorders given the association between activity, sleep, energy and mood. For example, Zak had a goal to improve his sleep and energy levels in order to boost his mood. Behavioural experiments allowed him to discover that activity gave him energy in the moment, directly lifted his mood and made him more tired for sleep later that night. Consequently, a daily 90 minute walk became an integral part of his recovery plan.

## 8. Reducing the impact of night time disturbance

‘*It's a policy that staff check on all patients through the night to ensure they are safe. Whilst we can't change this, we can think about how you would like them to be carried out so that they are less disruptive to your sleep*’

It is established policy that nursing staff regularly check on patients throughout the day and night to manage safety. The frequency of these are: hourly, every 15 minutes, or continuously, depending on risk assessment. They require the staff member to clearly see the patient breathing. At night this typically involves switching on the patient's bedroom light, opening the window hatch in the door and, if necessary, entering the bedroom.

Whilst necessary for patient safety, clearly such observations pose a significant challenge for sleep. This was addressed by acknowledging the associated frustration and committing to work with patients to minimize the disruption. Preferences were sought. For example, some patients chose the window hatch to be left open all the time (to reduce noise) whereas others preferred it to be shut between observations to maintain privacy. Similarly, some patients favoured having a dim light on throughout the night, whereas others preferred it to be switched on and off at each observation. Patients were also offered earplugs and eye masks (risk assessment permitting).

In addition to these strategies directly facilitating sleep, patients also appreciated ward staff accommodating their individual preferences. This may have reduced frustration and hence further promoted good sleep.

## 9. Working with distressing experiences at night

‘*Bed is a common place for worries to come to mind, or for hearing voices. If either of these are keeping you awake, we can work on them*’

The therapy was tightly focused on breaking maintenance factors for insomnia. Therefore if worry or voices were fuelling sleep difficulties, interventions were introduced to boost the effectiveness of the key CBT for insomnia techniques. If, however, these experiences were present, but not impacting on sleep, they were not addressed.

Worry that increases hyperarousal was very common in the patient group. The content varied, including concerns related to the experience of hospitalization and the wider impact on their life (e.g. relationships, study or work), treatment and the timing of discharge and also psychotic experiences (e.g. Billy's worries about people intending to attack him). The process of worry and the impact it had on sleep was targeted by integrating worry periods into the early evening routine, following a briefer form of the method used in Freeman et al. (2015a). For example, Billy used a 20 minute worry period at 6 p.m., where he wrote down his concerns and used problem solving to action those that he could influence. He shifted attention away from the worry after this 20 minute period by calling a family member. If worries came to mind when in bed, he used his pre-prepared cue card which reminded him to postpone the thought until the following day when he would be better placed to manage it (and hence keep bed as a worry-free zone). If worries continued to persist (and hence decrease the chances of sleep) he interrupted these by following the 15 minute rule.

Whilst a sub-sample of patients were experiencing voices which the therapists suspected were impacting on sleep, use of voice management techniques (e.g. using earplugs or postponing voices) were very limited. Some patients did not want to discuss voices because they were particularly distressing, others had concerns about the impact of discussions on the duration of admission. We suspect, however, that voices were being indirectly addressed via other sleep strategies. For example, the wind-down period decreased arousal and hence made it less likely that voices would be triggered by heightened affect. The 15 minute rule involves patients leaving bed and disengaging from internal experiences which increase arousal (whether that be voices, frustration or anxiety). Instead they shifted attention to wind-down techniques which commonly included relaxation audios. These provided an alternative auditory input and hence perhaps disrupted engagement with voices.

## 10. Managing discharge as a challenge for sleep

‘*What are the most useful things you've learnt over the course of therapy? How might these work when you're at home?*’

The process of discharge can bring advantages and disadvantages for sleep. It removes some of the challenges for sleep outlined previously (e.g. night-time observations) but can also be a time of stress. It might entail returning to a difficult environment or relationship, moving to new accommodation, or losing the structure and support available from hospital staff and therefore can be a test for the sleep system. Consequently throughout therapy, both ward and home sleep environments were considered and a dedicated session at the end of the 2-week window was used to develop a sleep plan for discharge.

The patient summarized their key learning from the therapy. They identified strategies to continue using upon discharge and any additional resources they would need to enable them to do so. A relapse management plan was developed to consider factors that led to the person's sleep difficulties previously, and how these could be managed in the future.

Billy was anxious about discharge and how he would manage at home. A detailed plan was therefore developed which included using alarm clocks (in place of staff prompts) to help him wake in the morning and continuing to ‘put the day to rest’ given his high levels of worry. Sandeep reported benefiting from the use of blackout blinds and hence his family purchased one for his home. Furthermore, he identified that an accumulation of life stressors had precipitated his sleep difficulties and admission, and so a plan was developed to minimize the impact of stress on sleep.

Once discharged, patients were offered a follow-up session in their home which allowed refinement of this plan and a chance to problem solve any difficulties that had arisen. Key individuals in the client's support system such as family members and/or the care coordinator were invited. The patient shared the pro-sleep strategies to promote a consistent approach to support. These meetings also offered a valuable opportunity to celebrate the positive changes the person made in relation to their sleep.

## Conclusions

Despite the challenges for sleep and the delivery of CBT which are inherent in an inpatient environment, the therapy described led to large effect size improvements in insomnia (Sheaves et al., 2017). Stabilizing sleep was a clear, specific and achievable target, amongst an often complex clinical picture. Core components of effective CBT for insomnia were delivered using creative adaptions to increase accessibility on the ward. These were augmented by novel technologies including sleep monitoring watches and light boxes to boost effectiveness for acutely unwell patients. Whist this work was at times challenging, the opportunity for creativity was exciting and the patient response and appreciation of therapy was extremely rewarding. The resource requirement for this therapy (such as daily psychological input) is a departure from traditional approaches and may require service level adaptations. However, given the potential clinical, service level and economic benefits, this approach warrants further investigation. The work has led to the formation of a new sleep clinic on the ward where the trial was based. Clinical staff offer sleep therapy to patients, using the treatment manuals developed within the trial, under the supervision of B.S. and L.I. Sleep medicine and its application to those experiencing psychiatric symptoms continues to be a fast-evolving field of research. We are eager to see how these developments continue to inform and refine this popular therapy.

## Supplementary material

For supplementary material accompanying this paper visit http://dx.doi.org/10.1017/S1352465817000789. click here to view supplementary material
