# Supplementary material for: Adapted CBT to Stabilize Sleep on Psychiatric Wards: a Transdiagnostic Treatment Approach
Source: Behav Cogn Psychother. 2018 Apr 4;46(6):661–75. doi: 10.1017/S1352465817000789 (PMC6141994; doi:10.1017/S1352465817000789)
Supplement: Supplementary file 1 [file S1352465817000789sup001.docx]

**Supplementary material:**

**
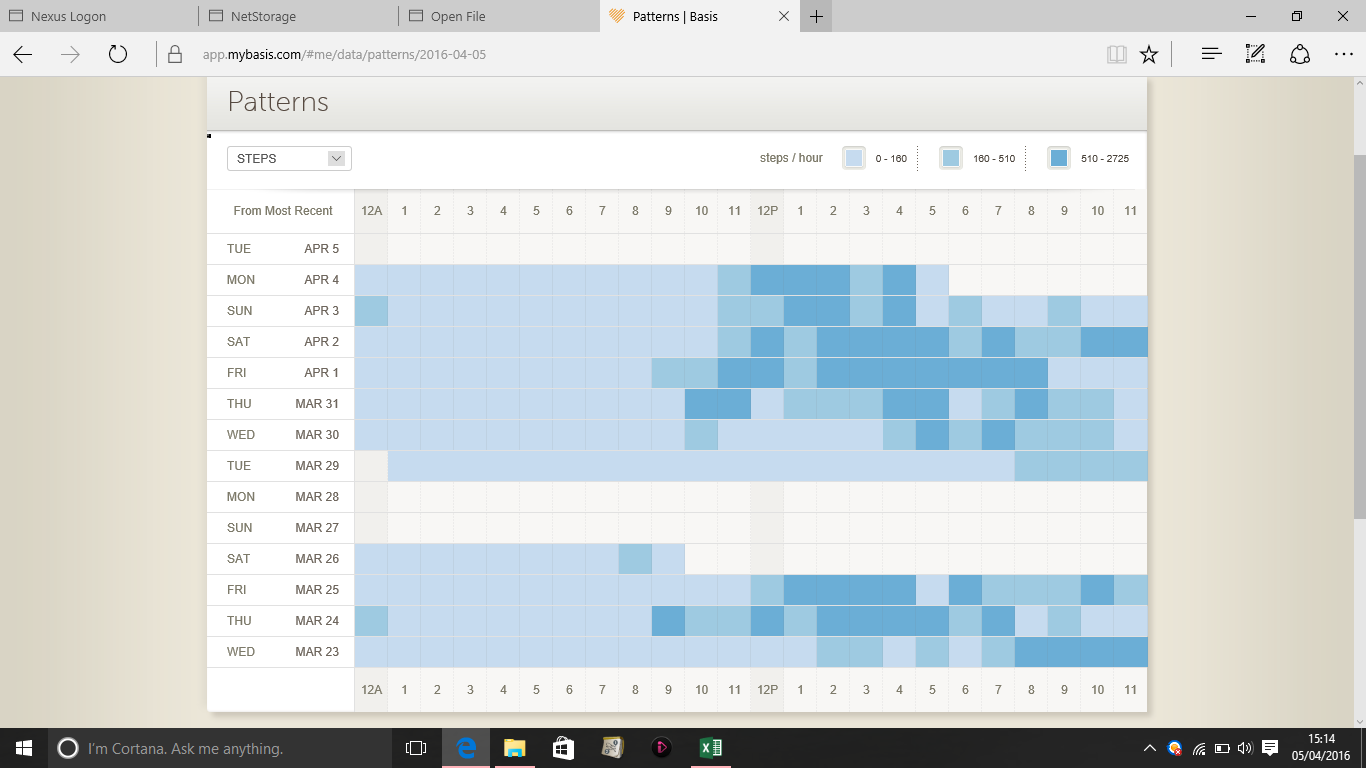
**

**Darker colours represent increased activity.**

**Figure 1. Basis Peak watch output of step count for assessment of circadian rhythms.**
